# Mimicking Bidirectional Inhibitory Synapse Using a Porous‐Confined Ionic Memristor with Electrolyte/Tris(4‐aminophenyl)amine Neurotransmitter

**DOI:** 10.1002/advs.202400966

**Published:** 2024-03-14

**Authors:** Kang Chen, Keyuan Pan, Shang He, Rui Liu, Zhe Zhou, Duoyi Zhu, Zhengdong Liu, Zixi He, Hongchao Sun, Min Wang, Kaili Wang, Minghua Tang, Juqing Liu

**Affiliations:** ^1^ School of Materials Science and Engineering Xiangtan University North Second Ring Road, Yuhu Xiangtan Hunan 411105 China; ^2^ Key Laboratory of Flexible Electronics (KLOFE) & Institute of Advanced Materials (IAM) Nanjing Tech University (NanjingTech) 30 South Puzhu Road Nanjing 211816 China

**Keywords:** bidirectional inhibitory synapse, ionic memristor, polyelectrolyte, porous polymer

## Abstract

Ionic memristors can emulate brain‐like functions of biological synapses for neuromorphic technologies. Apart from the widely studied excitatory‐excitatory and excitatory‐inhibitory synapses, reports on memristors with the inhibitory‐inhibitory synaptic behaviors remain a challenge. Here, the first biaxially inhibited artificial synapse is demonstrated, consisting of a solid electrolyte and conjugated microporous polymers bilayer as neurotransmitter, with the former serving as an ion reservoir and the latter acting as a confined transport. Due to the migration, trapping, and de‐trapping of ions within the nanoslits, the device poses inhibitory synaptic plasticity under both positive and negative stimuli. Remarkably, the artificial synapse is able to maintain a low level of stable nonvolatile memory over a long period of time (≈60 min) after multiple stimuli, with feature‐inferencing/‐training capabilities of neural node in neuromorphic computing. This work paves a reliable strategy for constructing nanochannel ionic memristive materials toward fully inhibitory synaptic devices.

## Graphical abstract

A bidirectional inhibitory synapse is presented in the vertical porous‐confined ionic memristor with electrolyte EV(ClO_4_)_2_ coated microporous tris(4‐aminophenyl)amine as neurotransmitter. The synaptic cell exhibits inhibitory plasticity under both positive and negative pulses due to porous‐confined ion migration for trapping and detrapping.

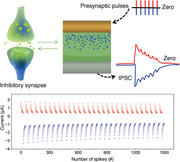

## 1 Introduction

Neuromorphic computing is a power‐efficient and high‐throughput computing paradigm inspired by biological neural system,^[^ ^1^, ^2^ ^]^ which offers a promising solution to the memory wall bottleneck faced by the conventional Von‐Neumann architecture‐based computers.^[^ ^3^ ^]^ Memristor is a more favorable candidate for the construction of neuromorphic computing system ascribed to intrinsic history‐dependent conductance modulation,^[^ ^4^ ^]^ which could emulate synaptic weights in biological neural networks.^[^ ^5^, ^6^ ^]^ Hitherto, remarkable advancements have been achieved in the innovation of synaptic memristors.^[^ ^7^, ^8^ ^]^ By reproducing the neuroplasticity such as short‐term potentiation (STP),^[^ ^9^ ^]^ long‐term potentiation (LTP),^[^ ^10^ ^]^ short‐term depression (STD) and long‐term depression (LTD),^[^ ^11^ ^]^ which represent temporal or sustained enhancement/decay of synaptic connections,^[^ ^12^ ^]^ the brain‐like learning and computing for the settlement of sophisticated tasks was feasible in them.^[^ ^13^ ^]^ As the opposite process of potentiation, STD and LTD were used to maintain the balance of depolarizing and hyperpolarizing membrane currents,^[^ ^14^, ^15^, ^16^ ^]^ which are fundamental for modeling sensory perception, learning, memory and forgetting.^[^ ^17^, ^18^ ^]^ Whereas, with respect to the extensive studies on those memristors with excitatory–excitatory or excitatory‐inhibitory features,^[^ ^19^, ^20^, ^21^, ^22^ ^]^ researches on the memristors exhibiting solely inhibitory synaptic behaviors remains elusive.^[^ ^23^ ^]^

Conjugated microporous polymers (CMPs) possess a distinctive combination of extended π‐conjugation and permanent microporous backbone.^[^ ^24^ ^]^ Their rich structural units and networks^[^ ^25^, ^26^ ^]^ give rise to diverse properties that find extensive applications in various microelectronics and optoelectronics such as thin‐film transistors,^[^ ^27^ ^]^ light‐emitting diodes^[^ ^28^ ^]^ and memristors.^[^ ^29^ ^]^ Currently, CMPs‐based memristors have surged in several intriguing applications. For instance, imine polymer thin‐film memristors grown at the gas‐liquid interface show reliable resistive switching at high temperature (340 °C) and in organic solvents due to their high conjugation degree.^[^ ^30^ ^]^ Light/steam cross‐linked porous amorphous polymer nanofilms serve as memristive materials that show remarkably long retention time and fast set (70 ns)/reset (845 ns) operations due to its microporous structure for efficient atom migration.^[^ ^31^ ^]^ Furthermore, carbazole‐based films incorporating an electron transfer system were prepared through a simple yet efficient electrochemical polymerization, resulting in an Al/CMPs/ITO memristor that exhibits a low turn‐on voltage of 0.35 V and a high switching ratio of 10^4^.^[^ ^32^ ^]^ By integrating a solid‐state electrolyte and CMPs to construct bilayer heterostructure, the micropore could serve as channels for confined and hysteretic ion transport, achieving ion memristive features with neuromorphic behaviors.

In this work, we first report a vertical porous‐confined ionic memristor with bi‐directional inhibitory synaptic function by combining solid electrolyte EV(ClO_4_)_2_ and microporous polymer tris(4‐aminophenyl)amine (TPA‐CMP) as neurotransmitter. The memristive and related synaptic properties were measured to associate bidirectional inhibitory capability. To unravel bidirectional inhibitory mechanism of this synapse, Brunauer–Emmett–Teller (BET) test and theoretic calculation are used to characterize the porous structure. By combing these structural and electrical analysis, a proposed mechanism of microporous‐confined ionic migration for nonvolatile ion‐trapping and detrapping was demonstrated. We also performed the training and inference operation in a small neural network where each node contains a bidirectional inhibitory synapse. The study provides an effective strategy to construct nanoionic systems for inhibitory neuromorphic hardware.

## 2 Results and Discussion

### 2.1 Structure and Function of Bidirectional Inhibitory Synapses

**Figure 1:**
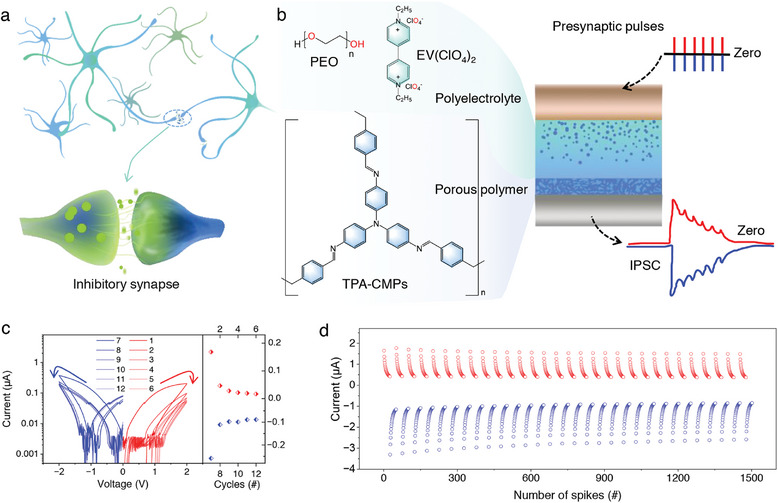
Synapse structure and function. a) shows biological neurons at the top and a inhibitory synapse at the junction of two nerve cells at the bottom with ions flow between cells. b) presents a schematic diagram of artificial synapse structure, the output is depressed under both positive and negative pulse stimuli. c) I−V curves of the device during six consecutive positive and negative sweeps, both showing a continuous drop in conductance. d) The device endurance test is evaluated by 30 periodic pulses. 25 pulses are of the same polarity.

**Figure** **1**a depicts a biological neuron with an enlarged view of the synapse (below) at the junction of two neurons, illustrating the cytokinesis of the neuron in response to stimulation. The balance of excitatory and inhibitory properties in the human body is a prerequisite for maintaining a healthy organism,^[^ ^16^, ^33^, ^34^ ^]^ while the plasticity of inhibitory synapses modulates the function of excitatory neural circuits and ultimately contributes to learning and memory as well as the refinement of neural circuits.^[^ ^35^ ^]^ A pure inhibitory synaptic diode with a two‐terminal structure was constructed by solid electrolyte and porous conjugate polymerization (Figure 1b, see details in the Experimental Section). The CMP film features a thin thickness of ≈15 nm with a low roughness of 0.53 nm (Figure S1, Supporting Information). The optical images of CMP film, solid electrolyte and their synapse were shown in Figure S2 (Supporting Information), revealing outstanding flatness. The transmission of neural spike signals carrying specific information from presynaptic to postsyaptic neurons in biological synapses is contingent upon the strength of synaptic connections.^[^ ^36^ ^]^ The movement of ions in the solid electrolyte specifically mimics inter‐synaptic cytokinesis, while the porous conjugated polymer functions as a transmitter‐gated ion channel. The fabricated synapse exclusively show inhibitory plasticity characteristics, generating postsynaptic currents that correspond to currents that correspond to the inward flow of chloride ions in the biological spike to produce hyperpolarization when stimulated with both positive and negative voltages. The modification of synaptic effects accompanied by successive DC voltage scans is illustrated in Figure 1c (also replotted in ordinary scale, Figure S3, Supporting Information). During the six consecutive clockwise forward DC voltage scans (0v‐2v‐0v), the peak conductance of the synaptic device gradually decreases to five times its initial value, indicating a gradual weakening of the synaptic connection. Subsequent six subsequent counter‐clockwise negative voltage scans (0v‐2v‐0v) showed the same trend, resulting in a sixfold reduction in peak conductance. The performance of devices based on other CMPs was also examined, however, resulting in a fall short of the anticipated level (Figure S4, Supporting Information). This bi‐directional suppression behavior of TPA‐CMP based device was also achieved with various scanning ranges such as 1, 1.25, 1.5, and 1.75 V (Figure S5, Supporting Information). The cyclic I‐V scanning curves were acquired at −3∼3, −4∼4, and −5∼5 V sweep ranges, all of which exhibited a consistent decline in conductance (Figure S6, Supporting Information). The manifestation of spike‐induced synaptic depression was displayed in Figure 1d, a sequence of 25 positive pulses (2 V, 50 ms) and negative pulses (−2 V, 50 ms) were applied to the synapses cyclically to regulate synaptic connectivity during the 30 cycles. The synaptic connectivity was assessed with a 0 V readout bias after each stimulation. A series of positive voltage spikes resulted in a significant fivefold reduction in synaptic weights, followed by a return to baseline by negative voltage spikes at each cycle, suggesting the successful synaptic weight updating. The synaptic device also exhibits outstanding cycling performance and endurance after undergoing 1500 weight changes. Following 30 cycles, its conductivity only experiences a marginal decrease of 6% with respect to the initial state.

**Figure 2:**
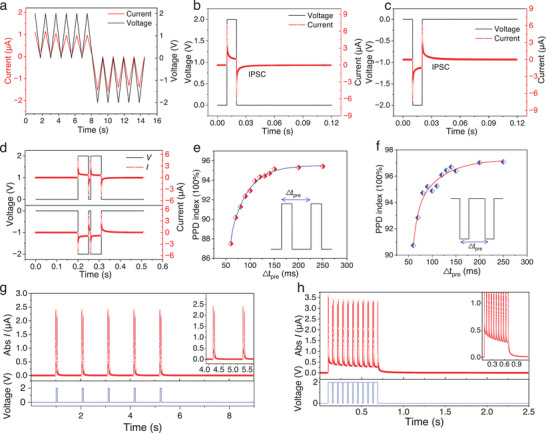
Electrical characterization of bidirectional inhibitory synapses. a) Current responses with time under bias pulses. (b) and (c) are the IPSC that are triggered with pulses of different polarity. d) Depression behaviors under positive and negative pulse pairs. PPD index of e) positive and f) negative pulses as a function of presynaptic pulse interval, showing more efficient modulation in shorter intervals. g) Short‐term depression (STD) with a spike interval of 1 s, long‐term depression (LTD) with a spike interval of 0.01s.

The memory behaviors of the device were investigated by subjecting it to various stimulation ways. The Short‐term depression (STD) characteristics of the device in response to positive and negative delta waves (2.0 V amplitude) were evaluated, which mimic synaptic weight variation observed in actual neural system under spike‐wave signals. The conductance of the device gradually decreases under a positive pulse (+2.0 V). Similarly, a negative pulse (−2.0 V) also leads a decrease of the conductance, demonstrating a bidirectional inhibition feature (**Figure** **2**a). The inhibitory post‐synaptic current (IPSC) was measured utilizing an electrical stimulus (±2 V, 10 ms) with a monitor bias of 0 V. Upon application of electrical pulse, there is an immediate increase in current followed by a gradual decrease throughout its duration. Then a current with opposite polarity emerging upon and subsequently decay over time upon withdrawal of the electrical pulse (Figure 2b,c). PPD is a foundational short‐term plasticity in biological synapse that means the current response of the second pulse is smaller than that of the first one. By measuring the weight change of two successively evoked postsynaptic signals, the PPD was also quantitatively evaluated both under positive and negative pulses (Figure 2d). The minimum PPD index of 87.50% and 90.74% under positive and negative electrical stimuli, respectively, occurs at a short interval of 50 ms. The decay curves were fitted with a double exponential function to obtain τ_1_ = 2.30 × 10^−3^ s and τ_2_ = 0.035 s for the positive electrical pulse, and τ_1_ = 8.96 × 10^−3^ s and τ_2_ = 0.054 s were obtained for the negative electrical pulse. The PPD index exhibits an increase as the interspike interval lengthens, indicating a decline in synaptic transmission efficiency. When the interval was extended to 250 ms, the inhibition effect weakened and resulted in a rise of the positive PPD index from 87.50% to 95.45% (Figure 2e) and the negative PPD index from 90.74 to 97.09 (Figure 2f). The aforementioned observation implies that increased frequency of inputs can result in accelerated rates of “forgetting”. Short‐ term plasticity refers to a transient deformation of synaptic function, leading to a temporary loss of information within a neuron. Conversely, long‐term plasticity denotes a lasting alteration in synaptic connectivity. The transition from STD to LTD plays a pivotal role in the psychological memory and forgetting models within biological brain, which is mimicked by simulating synapse with electrical pulses with the same amplitude and duration, but at different intervals. In Figure 2g, a single stimulus (+2 v,50 ms) with 1 s intervals shows a short‐lived inhibition. The synaptic weights quickly returned to the original state and had no effect on subsequent synaptic weights. By contrast, repeated activation of frequent stimuli resulted in enduring inhibition of synaptic weights (Figure 2h). Similar results were observed during negative electrical pulse measurement, Figure S7 (Supporting Information).

### 2.2 Bidirectional Modulation of Inhibitory Plasticity

**Figure 3:**
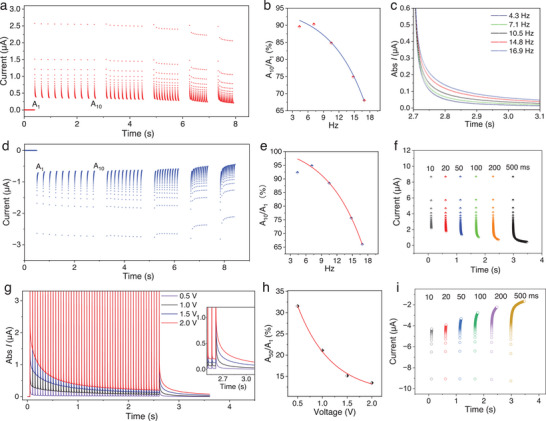
Synaptic plasticity. (a) and (d) show the characteristics of spike rate‐dependent plasticity (SRDP) under positive and negative pulses, respectively. b) and e) show the rate of conductance change for 10 spikes. c) shows the after‐current generated by ten different frequency pulse stimuli, with more significant inhibition at higher frequencies. f) shows the inhibition effect with a single positive pulse. g) shows the inhibition effect at long times with pulse widths of 50 ms at 0.5, 1.0, 1.5, and 2.0 V and h) shows the change of A_50_/A_1_ conductance with several intensity pulses. i) presents the dependence of pulse width characteristics under negative pulses.

Spike rate‐dependent plasticity (SRDP) is considered as one of the fundamental forms of synaptic plasticity,^[^ ^37^ ^]^ wherein the regulation of synaptic weights occurs in response to variations in spike rate. The current response to stimulation at five different frequencies (4.3, 7.1, 10.5, 14.8, and 16.9 Hz) is displayed in **Figure** **3**a, with each pulse width fixed at 50 ms and the intensity at +2 V. Apart from the first pulse deviation, higher frequencies exhibit superior inhibition. For the last four frequency sets, the rate of conductance change of the ten spikes decreased exponentially as the input signal interval was shortened (Figure 3b). The postcurrents generated after ten pulses were recorded at five frequency sets. The higher the frequency, the greater the postcurrent, indicating a significant enhancement in synaptic weight (Figure 3c). A similar scenario occurred under negative electrical pulse stimulation (Figure 3d,e). In addition, the duration of the stimulus is also a crucial factor that influences the alteration in synaptic weights. The impact of pulse width on organic synaptic weights was investigated by varying the pulse width from 10 to 500 ms. The inhibitory effect was significantly enhanced with increasing pulse width, and it was clearly observed that the current continued to decrease with the pulse stimulation at a width of 500 ms. (Figure 3f for the positive pulse and Figure 3i negative phase pulse). In biological synapses, stronger stimulation of the preceding neuron is observed as a more pronounced change in current in the next neuron. Figure 3g illustrates the dependence of weight change on pulse amplitude. For 50 consecutive 50 ms pulse stimuli, the ratio of A_50_/A_1_ decreases continuously with increasing intensity, with a value of 31.55% at 0.5 V pulses, yet only 13.49% at 2 V, suggesting a distinct intensity‐dependent property of artificial synapse (Figure 3h). Spike time‐dependent plasticity (STDP) is a significant learning mechanism in the biological brain, playing a fundamental role in the processes related to learning and memory. It describes the phenomenon where the pre‐synaptic neuron is stimulated earlier than the postsynaptic neuron (dt > 0), leading to an enhancement of postsynaptic current. Conversely, when the presynaptic neuron is stimulated after the postsynaptic neuron (dt < 0), it results in inhibition of postsynaptic current. Additionally, altering the time difference between these two stimuli also impacts the extent of enhancement observed in the postsynaptic current. To quantify this effect, we define weight changes as △W = (A_2_‐A_1_)/A_1_*100%. Figure S8 (Supporting Information) depicts typical STDP behavior obtained by varying dt values. Furthermore, the environmental stability of the device was evaluated by performing DC scan and pulse tests after being exposed to air for 150 days, revealing no discernible difference with respect to the initial preparation (Figure S9, Supporting Information), thus demonstrating excellent environmental stability.

### 2.3 Mechanism of Bidirectional Inhibitory

**Figure 4:**
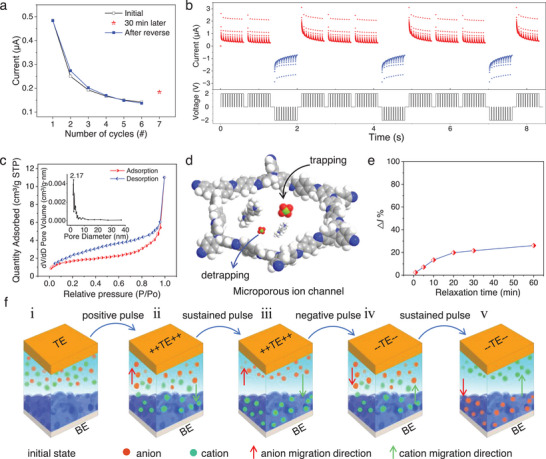
Bidirectional inhibition mechanisms. a) Variation trend of DC scanning current under different conditions. b) The inhibitory effect of a series of alternating positive and negative presynaptic stimuli. Note that the positive pulse can return to the initial state when the negative‐phase pulse stimuli is followed by the positive pulse. C) Isothermal adsorption curves of CMPs N_2_ tested at 473.15 k. The inset shows the estimated pore size distribution based on the BJH equation. d) The pore size of the film under theoretical conditions, with indication of ion migration through the film. e) Increase in current at different resting times after six inhibitions. f) ion transport storage mechanism in solid electrolyte and TPA‐CMPs functional layer.

The variation of the peak current of the device for three scans under forward DC bias is illustrated in **Figure** **4**a. The initial state is represented by black line, and as the number of scans increases, the peak current decreases from 0.48 to 0.14 µA after six scans. Even after a period of inactivity lasting 30 min, the conductance state does not return to the initial state (red star). However, after six negative scans, the device is able to return to the initial state, and the peak current after each scan aligns well with the original data (blue line), which underscores the non‐volatile nature of our device. The conductance of ionic memristor is influenced by scanning rate and temperature. When subjected to positive and negative DC voltage scans, the hysteresis window expands in proportion to the increase in scanning rate, leading to an enhancement in conductivity. Higher scanning speeds result in reduced energy transmission by the device and a decrease in inhibitory effects. (Figure S10, Supporting Information). The variable temperature I‐V curves further demonstrate a temperature‐dependent behavior, where the conductance exhibits an increase with rising temperatures, which is characteristic of typical ionic transporting properties, which is typical of ionic memristor characteristics (Figure S11, Supporting Information). Short‐term suppression was achieved by applying a series of 10 pulses with an intensity of 2 V, a width of 50 ms, an inter‐pulse interval of 10 ms, and a group interval of 100 ms (Figure 4b). Sustained inhibition was achieved by three consecutive sets of stimuli with the same pulse polarity, regardless of interval length. In groups of ten consecutive pulses, the current of the last pulse of each group was higher than the current of the first pulse of the latter group, indicating attenuated synaptic transmission after completion of the depressive process. These findings confirm that the organic electrochemical device is a highly mimetic and self‐regulating synapse. Interestingly, through sustained inhibition during negative pulse stimulation, a reconfiguration is achieved that allows for self‐resetting. Following a series of negative pulse stimulations, the excitation efficiency is fully restored compared to its initial state when crossed under DC scanning. This bidirectional inhibition of tunable synaptic properties validates the operating principle of our neuromorphic design. In both the TPA‐CMPs alone and the pure solid electrolyte structure, no continuous conductance changes were observed (Figure S12, Supporting Information).

To substantiate the hypothesis that the porous structure of the CMPs plays a crucial role, we conducted an investigation into the porous structure of TPA‐CMPs with nitrogen adsorption isotherms at 473.15 K (Figure 4c). The pore size was estimated via a Barret–Joyner–Halenda (BJH) model,^[^ ^38^ ^]^ revealing an optimal value of 2.17 nm, thereby providing exceptional ion channels for both anions and cations within the electrolyte (inset in Figure 4c). Figure 4d shows the migration of anions and cations in the pore channels. The dynamics of ions within the porous polymer was further investigated by performing six DC scans, followed by analysis of the resting time magnitude (Figure 4e). The gradual increase in device conductivity with extended resting time is attributed to the detrapping of ions from the CMPs and their subsequent detention by the solid electrolyte, resulting in enhanced current flow due to a higher concentration of mobile ions within the system. Whereas, the response exhibit limitations as the current only increases by 26.1% after 1 h rest and fails to return to its initial state, indicating its non‐volatile feature. Based on the above experimental results, we propose a possible bidirectional inhibition mechanism of artificial synapses (Figure 4f), **i)** the anions and cations in the solid‐state electrolyte are randomly and uniformly distributed in the absence of an external electric field. **ii)** When a forward bias or pulse is applied to the top electrode, the anions and cations in the electrolyte migrate directionally, with the anions converging toward the top Au electrode and the cations being doped into the porous CMPs layer. Due to binding with the CMPs layer, short term return of migrated ions to the solid electrolyte becomes challenging,^[^ ^39^ ^]^ resulting in a decrease in freely mobile ions within the system. The limited increase in conductivity of the insulating CMPs due to doping leads to a continuous decline in conductivity that is hindered by synaptic weights reduction. After prolonged inhibition, some ions migrate back into the electrolyte, causing a slight increase in conductivity but not returning to the initial state, corresponding to Figure 4e. After multiple scans, the majority of mobile ions are distributed at the Au electrode interface as well as in the CMPs layer (**iii**) where the conductivity of the device does not decrease further. However, when opposite polarity scans or pulses occur, the free ions are aggregated at the Au electrode and bound in the CMPs (**iv**), resulting in peak conductivity during the first reverse followed by a gradual decrease in ion migration similar to that observed during the forward scan.

**Figure 5:**
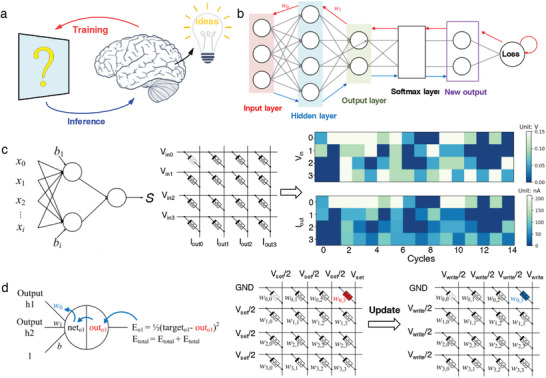
a) A image of the human brain illustrates the processes of training and inference. b) Schematic of neural network structure. c) Schematic of inference operation. d) Schematic of training operation.

Human beings gather physical information about the external world through various senses and then transmit it to the cerebral cortex for perception and learning. **Figure** **5**a shows how the human brain processes information from the outside world to get the results it wants. Utilizing our fully inhibitory synapses, we constructed a small neural network and implemented a complete training and inference flow (Figure 5b). The blue pathway represents the inference operation, where input data is processed through each layer of the network to perform calculations and conversions, ultimately producing predicted outputs. The red pathway represents the training operation, which involves evaluating errors between predicted results and true values, calculating errors for each neuron layer using chain rule methodology, and updating weight parameters. Although we prepared a bidirectional inhibitory synapse, it is not only the reduction of weights but also the updating of weights that is achieved under continuous DC scanning or pulsing (Figure 4a,b). For example, the minimum conductance was reached under ten positive pulses, and the highest conductance (with the largest weights) was reached in the first pulse after the polarity reversal of the pulse (Figure 4c). In this 4 × 4 neural network array, each node functions as a fully inhibitory synapse. During the inference process, the trained weight parameters are stored in the memristors, and the feature vector is converted into corresponding voltage values, which are then input to their respective memory rows for performing inference operations. Figure 5b illustrates the eigenvector input and inference output of 16 operations conducted by this array. On the other hand, the weights of each node in this neural network are updated through training operations (Figure 5d). Weight training primarily consists of the two steps: weight erasing and weight writing. During the training phase, the voltage in the row of the selected cell is grounded, while the voltage in the column is set to −2 V (V_reset_) for erasing the existing weight state. To write a new weight, the voltage in the row of the memory cell is grounded, and a continuous pulse voltage of 2 V (V_write_) is applied to the column for writing the required weight state onto the synapse, completing the weight update. It should be noted that other rows and columns are set to −1 V (V_set_/2) or 1 V (V_write_/2) respectively, ensuring no alteration of weight states in other memristor cells during weight updates.

## 3 Conclusion

In summary, we have demonstrated an ionic memristor that exhibits bidirectional inhibitory synaptic plasticity function. The target device features a vertical sandwich configuration with solid electrolyte as ions reservoir and microporous polymer bilayer as transport channels. The nanoslit structure of the bilayer enables confined migration, trapping, and detrapping of ions, resulting in distinctive bidirectional inhibitory plasticity in response to different polar electrical stimuli, and a long‐term memory level up to 1 h under repeated electrical pulses. Utilizing this inhibitory synapse as the neural node, we have achieved the fundamental training and inference functions for neuromorphic computing. We anticipate that this demonstration will inspire further investigation into bionic inhibitory synapse systems involving ions synaptic materials and devices.

## 4 Experimental Section

### Materials

Tris(4‐aminophenyl)amine and 1,4‐Phthalaldehyde were purchased from Energy Chemical. PEO, EV(ClO_4_)_2_, acetonitrile were purchased from Aladdin. The above raw materials were used directly without further purification.

### Device Fabrication

The neurosynaptic device consists of the following parts: ITO glass as the bottom electrode, TPA‐CMPs functional layer, PEO‐EV(ClO_4_)_2_ solid electrolyte ion transport layer, and Au top electrode. The ITO glass were cleaned with deionized water, ethanol, and isopropanol for 15 min by ultrasonication and then blown dry with a nitrogen gun. Tris(4‐aminophenyl)amine and 1,4 Phthalaldehyde DMF precursors were prepared in glass bottles of 0.05 and 0.075 mmol mL^−1^, respectively. The TPA‐CMPs active layer was prepared by spinning 70 µL of the precursor mixture at 1000 rpm for 15 s and then at 1500 rpm for 50 s, spin‐coating onto an ITO glass substrate, and then reacting at 200 °C for three days. To obtain solid electrolyte solution, a 4 wt.% EV(ClO_4_)_2_ acetonitrile solution was prepared, followed by 1 mL of a 5 wt.% PEO acetonitrile solution. Then the prepared solution was filtered through a polytetrafluoroethylene membrane microfilter with a pore size of 0.45 µm to remove any undissolved particles, and 400 µl of EV(ClO_4_)_2_ solution was added to the filtered PEO acetonitrile solution to obtain the solid electrolyte precursor. The solid electrolyte layer was obtained by dropping 20 µL of electrolyte solution on the ITO glass with a well‐grown TPA‐CMPs layer and drying at 60 °C for 30 min. Finally, the devices were fabricated by thermal evaporation deposition of 100 nm thick Au electrodes through metal shielding under reduced pressure (10^−5 ^Pa).

### CMPs Pore Size Testing

The BET test was performed using a Micromeritics ASAP 2460, and the pore size data were obtained from the adsorption desorption curve of nitrogen at 200 °C.

### Characterization of Film Morphology

Photomicrographs of CMP, solid‐state electrolytes, and devices were made using a Nikon LV100ND, and CMP film thickness roughness using a Dimension ICON AFM.

### Electrical Measurement

DC and pulse characteristics of devices measured with Keithley 4200 SCS.

## Conflict of Interest

The authors declare no conflict of interest.

## Supporting information

Supporting Information

## Data Availability

The data that support the findings of this study are available from the corresponding author upon reasonable request.
