# Supplementary material for: Mimicking Bidirectional Inhibitory Synapse Using a Porous‐Confined Ionic Memristor with Electrolyte/Tris(4‐aminophenyl)amine Neurotransmitter
Source: Adv Sci (Weinh). 2024 Mar 14;11(19):2400966. doi: 10.1002/advs.202400966 (PMC11109647; doi:10.1002/advs.202400966)
Supplement: Supplementary file 1 — Supporting Information [file ADVS-11-2400966-s001.pdf]

ADVANCED SCIENCE
Open Access

Supporting Information

for *Adv. Sci.*, DOI 10.1002/advs.202400966

Mimicking Bidirectional Inhibitory Synapse Using a Porous-Confined Ionic Memristor with Electrolyte/Tris(4-aminophenyl)amine Neurotransmitter

Kang Chen, Keyuan Pan, Shang He, Rui Liu, Zhe Zhou, Duoyi Zhu, Zhengdong Liu, Zixi He, Hongchao Sun, Min Wang, Kaili Wang, Minghua Tang* and Juqing Liu**

Supporting Information

Mimicking bidirectional inhibitory synapse using a porous-confined ionic memristor with electrolyte/tris(4-aminophenyl)amine neurotransmitter

Kang Chen1, Keyuan Pan2, Shang He1, Rui Liu1, Zhe Zhou2*, Duoyi Zhu2, Zhengdong Liu2, Zixi He2, Hongchao Sun2, Min Wang2, Kaili Wang2, Minghua Tang1*, Juqing Liu2*

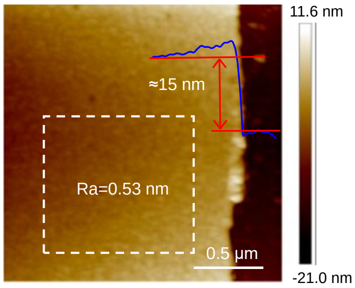

Figure S1. AFM diagram of TPA-CMP thin film

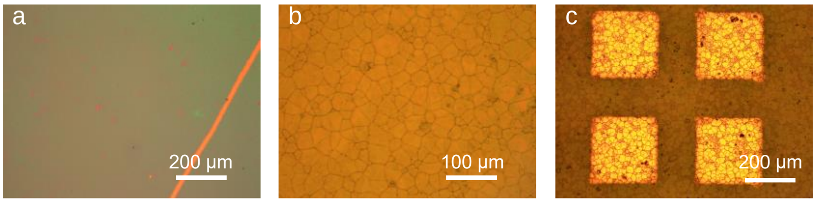

Figure S2. a-b) Optical image of CMP film and solid electrolyte, c) morphology and structure of the device

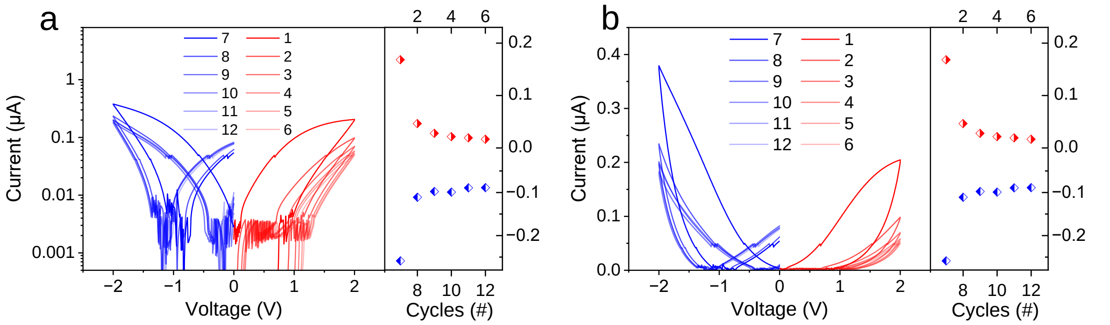

Figure S3. I–V curves of the device during 6 consecutive positive and negative sweeps, a) log scale b) ordinary scale.

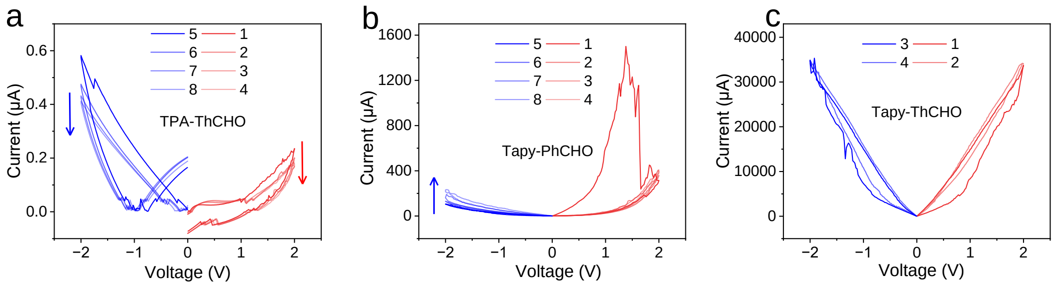

Figure S4. Initially, four distinct types of CMP materials, including Tris(4-aminophenyl)amine (TPA), 5,10,15,20-tetrakis(4-aminophenyl)porphyrin (Tapy), 1,4-phthalaldehyde (PhCHO), and 2,5 Thiophenedicarboxaldehyde (ThCHO), were synthesized for device preparation. a-c) The scanning I-V curves of TPA-ThCHO, Tapy-PhCHO and Tapy-ThCHO, respectively

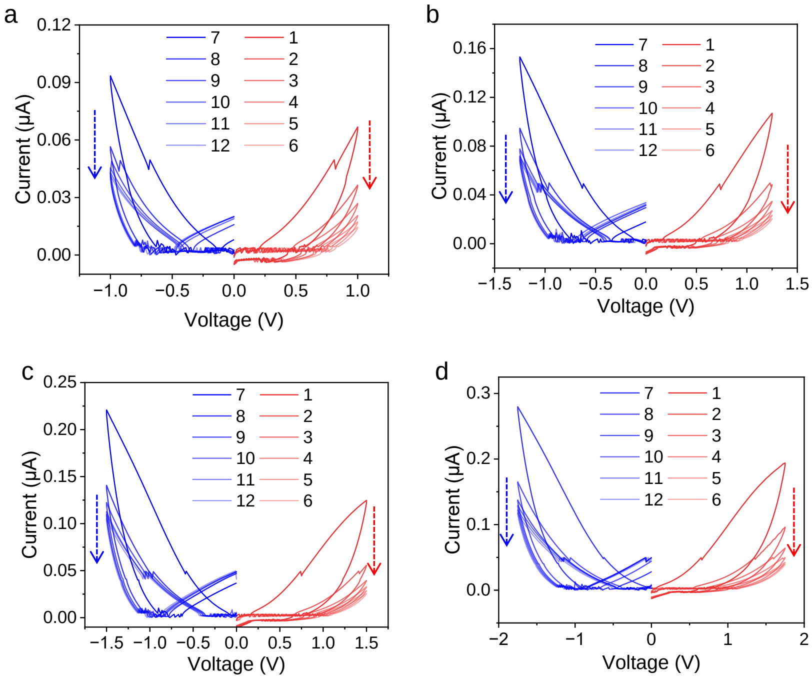

Figure S5. The DC scan curves of the devices at 1 , 1.25 , 1.5 , and 1.75 V demonstrate a decrease in conductance under both positive and negative scans.

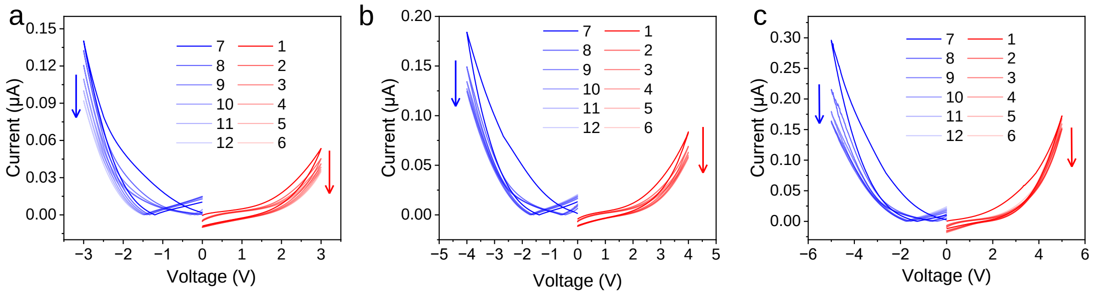

Figure S6. The DC scan curves of the devices at 3 , 4 and 5 V demonstrate a decrease in conductance under both positive and negative scans.

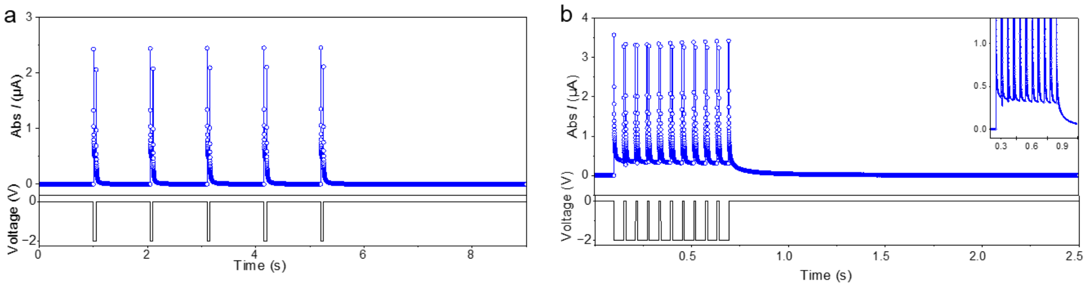

Figure S7. a) The spike interval for short-term depression (STD) with a -2v pulse is 1 second, and b) the spike interval for long-term depression (LTD) is 0.01 second.

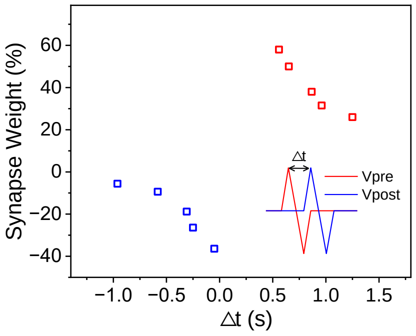

Figure S8. Learning rules for STDP of the memristor.

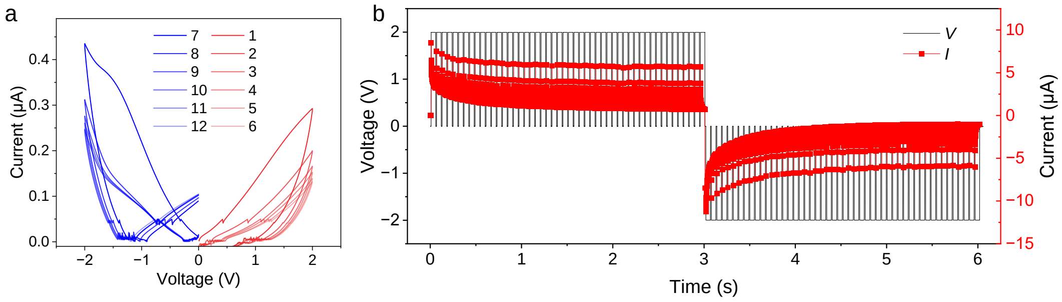

Figure S9. a) IV curve of the device after 150 days in air, and b) 50 consecutive +2V pulses followed by 50 pulses of -2V stability test.

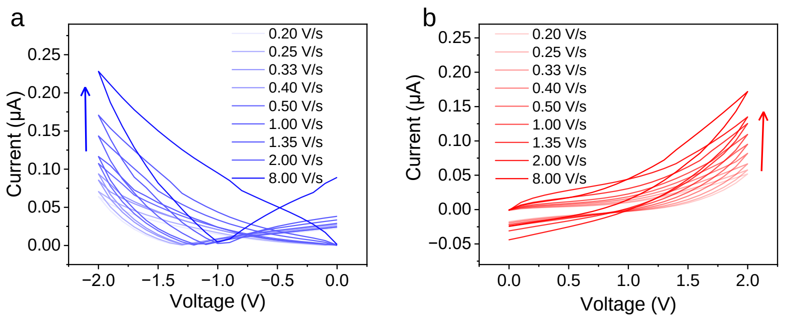

Figure S10. a-b) I-V curves of the device at different scan rates for negative and positive voltage

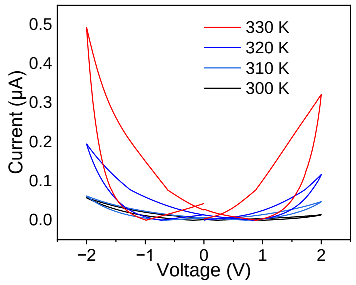

Figure S11. I-V curves of devices at different temperatures

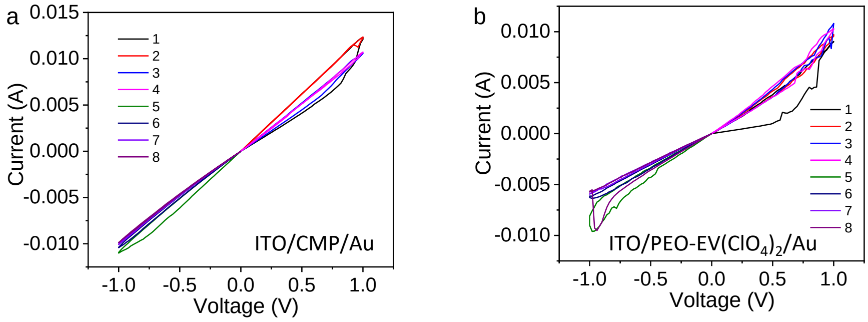

Figure S12. a) IV curves for devices with CMPs as the functional layer, b) IV plots for those with solid electrolytes as the functional layer.
